# Maximising Acute Kidney Injury Alerts – A Cross-Sectional Comparison with the Clinical Diagnosis

**DOI:** 10.1371/journal.pone.0131909

**Published:** 2015-06-30

**Authors:** Simon Sawhney, Angharad Marks, Tariq Ali, Laura Clark, Nick Fluck, Gordon J. Prescott, William G. Simpson, Corri Black

**Affiliations:** 1 University of Aberdeen Applied Renal Research Collaboration, Aberdeen, United Kingdom; 2 NHS Grampian, Aberdeen, United Kingdom; 3 King Faisal Specialist Hospital and Research Centre, Riyadh, Saudi Arabia; The University of Tokyo, JAPAN

## Abstract

**Background:**

Acute kidney injury (AKI) is serious and widespread across healthcare (1 in 7 hospital admissions) but recognition is often delayed causing avoidable harm. Nationwide automated biochemistry alerts for AKI stages 1-3 have been introduced in England to improve recognition. We explored how these alerts compared with clinical diagnosis in different hospital settings.

**Methods:**

We used a large population cohort of 4464 patients with renal impairment. Each patient had case-note review by a nephrologist, using RIFLE criteria to diagnose AKI and chronic kidney disease (CKD). We identified and staged AKI alerts using the new national NHS England AKI algorithm and compared this with nephrologist diagnosis across hospital settings.

**Results:**

Of 4464 patients, 525 had RIFLE AKI, 449 had mild AKI, 2185 had CKD (without AKI) and 1305 were of uncertain chronicity. NHS AKI algorithm criteria alerted for 90.5% of RIFLE AKI, 72.4% of mild AKI, 34.1% of uncertain cases and 14.0% of patients who actually had CKD.The algorithm identified AKI particularly well in intensive care (95.5%) and nephrology (94.6%), but less well on surgical wards (86.4%). Restricting the algorithm to stage 2 and 3 alerts reduced the over-diagnosis of AKI in CKD patients from 14.0% to 2.1%, but missed or delayed alerts in two-thirds of RIFLE AKI patients.

**Conclusion:**

Automated AKI detection performed well across hospital settings, but was less sensitive on surgical wards. Clinicians should be mindful that restricting alerts to stages 2-3 may identify fewer CKD patients, but including stage 1 provides more sensitive and timely alerting.

## Introduction

Acute kidney injury (AKI) is a serious, widespread and costly condition, usually diagnosed from rapidly changing blood tests (serum creatinine)[1]. It complicates 1 in 7 hospital admissions across a wide range of specialties[2]. The burden to NHS England is estimated at £1 billion/year or 1% of the NHS budget[2]. Mortality is high in severe AKI (up to 1 in 3)[3], but even small changes in creatinine are associated with a poorer prognosis[4].

AKI is often poorly recognised and inadequately managed. A recent UK confidential enquiry into patient outcome and death found that of individuals that died with AKI, 43% of those that developed AKI during hospital admission were recognised late, 22% of AKI patients received inappropriate management and 17% had avoidable complications[5].

Novel approaches have been developed to improve AKI recognition, understanding and reduce avoidable harm. In March 2015, NHS England introduced a mandatory nationwide system of automated AKI detection in hospitals. This system can “alert” clinicians when AKI is apparent on routine biochemistry tests[6]. It is based on internationally agreed Kidney Disease: Improving Global Outcomes (KDIGO) criteria[1]. Changes in serum creatinine are tracked in a biochemistry system with each new (index) test automatically compared to previous (reference) results. An AKI “alert” of severity stages 1–3 is generated if sufficient change in creatinine has occurred in a short space of time based on one of three criteria outlined in Table 1[6].

**Table 1:** Summary of NHS England AKI algorithm.

AKI Criteria	Definition for AKI (one of the three)
Criterion 1	Serum creatinine ≥1.5 times higher than the median of all creatinine values 8–365 days ago
Criterion 2	Serum creatinine ≥1.5 times higher than the lowest creatinine within 7 days
Criterion 3	Serum creatinine >26 μmol/L higher than the lowest creatinine within 48 hours
AKI Stage	Classification Requirements
Stage 1	Rise in creatinine of >26 μmol/L; or index/reference ≥1.5 and <2
Stage 2	Index/reference ≥2 and <3
Stage 3	Index/reference ≥3; or ≥1.5 and index creatinine >354 μmol/L (or 3 times the upper reference interval if age <18)

AKI can be diagnosed if any one of three criteria are met. Staging is based on a comparison of a serum creatinine (index) with a reference test. Where a creatinine is outside the reference range but a previous creatinine within one year is unavailable, the test is “flagged” abnormal (with chronicity unknown).

AKI can occur in any clinical setting (medicine, surgery, community, critical care), but an AKI alert may require a different interpretation in these different settings. Renal impairment may either develop suddenly (AKI), or it may be long-standing (chronic kidney disease, CKD). It is easier to judge the rapidity of these changes in patients with multiple morbidities or in a highly monitored environment because they will have more frequent and more recent blood tests available. In contrast, a patient with no previous illnesses may have had few infrequent blood tests or perhaps none. The rest of their clinical history may help an experienced clinician decide between a diagnosis of AKI or CKD, but an automated detection system can only categorise according to its pre-programmed criteria. This might limit the utility of automated detection to provide timely reliable alerting to AKI patients in some clinical circumstances.

In comparison, in a study by Ali *et al*., large scale case note review was performed on all patients in a regional population who developed significant renal impairment over a six month period in 2003[7]. This enabled the authors to distinguish AKI from CKD with the benefit of clinical records as well as biochemistry, and also to determine the cause of AKI and clinical setting. The authors used the older “Risk Injury Failure Loss End-stage” (RIFLE) criteria to diagnose AKI from which criterion 3 of the NHS England and KDIGO criteria is notably absent[8]. Patients without RIFLE AKI may therefore still have KDIGO AKI due to small absolute creatinine rises. The authors categorised such small rises in creatinine that did not meet RIFLE criteria separately as a “mild AKI” group.

Here we have used this well described cohort to compare automated AKI detection with the judgement of an experienced nephrologist reviewing both biochemistry and clinical case notes. All patients in the cohort had renal impairment, but with a mixture of AKI, CKD and impairment of uncertain chronicity across a range of clinical settings. The cohort represents a useful opportunity to explore how automated detection classifies AKI and CKD in a large but well characterised population with renal impairment in different clinical settings.

## Methods

### Study Population

**Fig 1:**
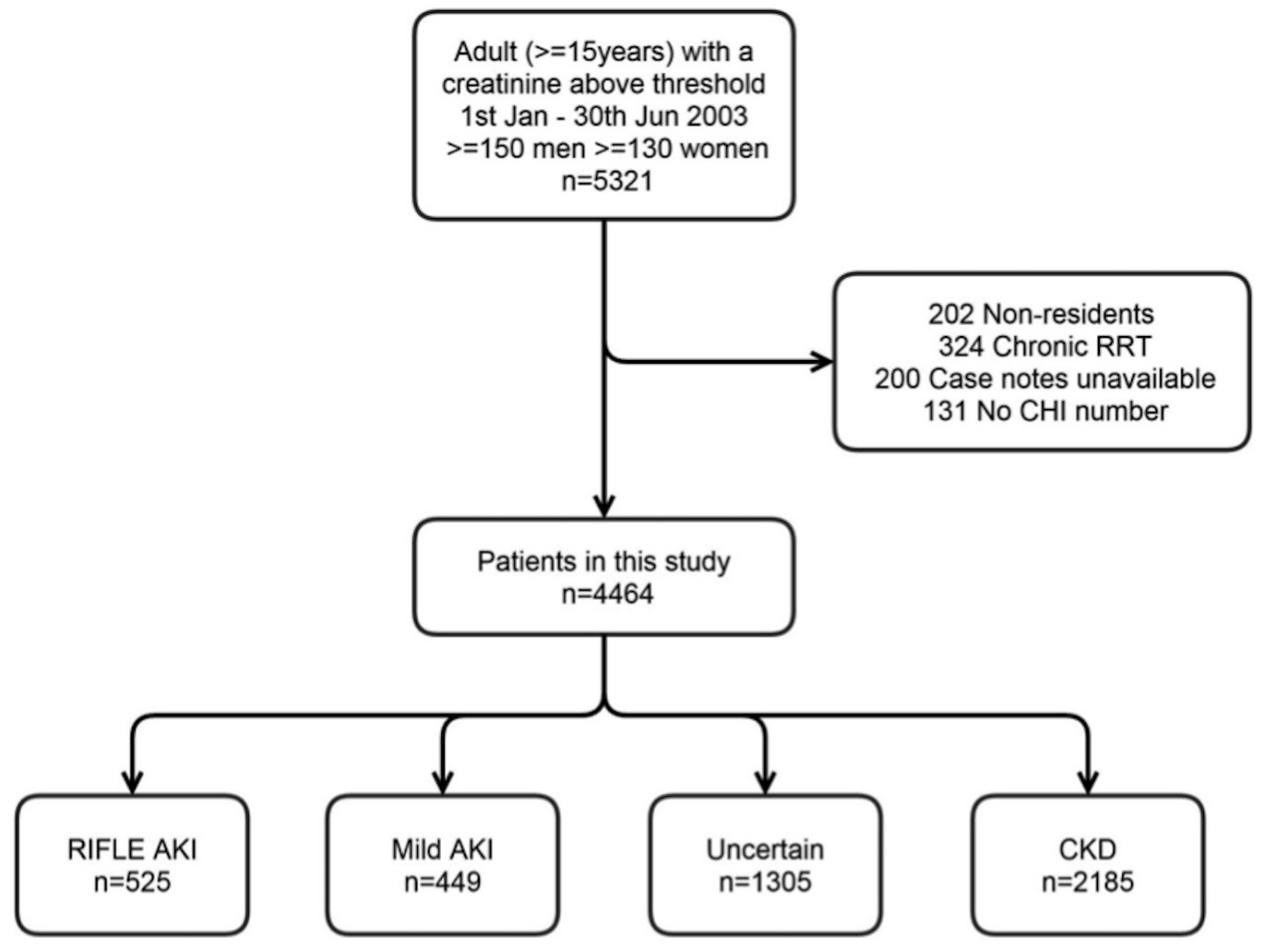
Study population. AKI = acute kidney injury, CHI = community health index, CKD = chronic kidney disease, RIFLE = risk injury failure loss end stage kidney disease, RRT = renal replacement therapy.

We used a previously well described cohort in the Grampian region of Scotland (adult population 438,332 in 2003)[7]. The cohort comprises all 5321 patients with at least one “abnormal” serum creatinine above a “threshold value” (150 μmol/l men, 130 μmol/l women) between 1^st^ January- 30^th^ June 2003. We excluded from this cohort patients who lived outside of Grampian, patients already receiving chronic renal replacement therapy (RRT), patients without case notes available, and patients without a community health index (CHI) number—a unique identifier for all residents in Scotland. 4664 patients were included in the final study (Fig 1). This study, including the use of anonymised data without formal patient consent, was specifically approved by NHS Grampian Research and Development, University of Aberdeen College Ethics Review Board and the Scottish Renal Registry. The Local Research Ethics Committee waived the need for their formal approval. Anonymised data for analysis were hosted and managed by Grampian Data Safe Haven.

### Data Linkage

We linked this cohort to the Grampian Regional Biochemistry Database. The Grampian biochemistry service processes all blood samples in the region regardless of clinical setting (hospital or community). In 2003 this included a large 900 bed hospital (Aberdeen Royal Infirmary) with intensive care, renal and cardiothoracic units; and two smaller outlying hospitals comprising a total of approximately 500 beds covering medicine, surgery, elective orthopaedics and medicine for the elderly. All serum creatinine values were isotope dilution mass spectrometry (IDMS) aligned. Matching on the CHI number resulted in biochemical profiles for all patients with measured creatinine values between 1999 and 2009.

### Clinical Reference Groups—Nephrologist Diagnosis of AKI and CKD

All patients in the cohort had renal impairment. The cohort was previously developed by Ali *et al*., by extensive review of the laboratory results and case-notes of every patient in the cohort[7]. In this previous study, patients were assigned to one of five categories, AKI, acute on chronic renal failure (ACRF), mild AKI, CKD, uncertain chronicity. The AKI definition was based on the creatinine changes in the “Risk Injury Failure Loss End-stage” (RIFLE) criteria which involved a change in creatinine or estimated glomerular filtration rate (eGFR)(Table 2)[8]. The RIFLE urine output criteria were not included. It should be noted that the current KDIGO definition now includes smaller rises in creatinine (≥26.4 μmol/l) than were included in the RIFLE definition (a ≥1.5-fold rise). CKD criteria were based on National Kidney Foundation Kidney Disease Outcomes Quality Initiative guidelines requiring persistent renal impairment for more than 3 months[9]. We used the same patient categories as were previously assigned by Ali *et al*. and these are outlined in Table 2. We modified the original categories in one respect by combining “AKI” and “ACRF” into one category—Any patients with AKI and CKD (ACRF) were included in the AKI group; and no patients with any changes suggestive of AKI were included in the CKD group.

**Table 2:** Nephrologist clinical diagnosis groups.

Clinical Group	Group Criteria
RIFLE AKI*	Risk (R)– ≥1.5-fold creatinine rise from baseline; or eGFR reduced by 25%.
	Injury (I)– ≥2 times-fold creatinine rise from baseline; or eGFR reduced by 50%.
	Failure (F)– ≥3 times-fold creatinine rise; or eGFR reduced by 75%; or a rise of ≥44 μmol/l to a creatinine above 350μmol/L.
	Baseline value in the previous six months, or alternatively a corresponding fall in creatinine in the subsequent six months if a previous baseline was unavailable.
CKD	Three previous tests greater than or equal to the “threshold value” each at least one month apart from 1st January 1996 onwards. A cluster of results above the “threshold value” that did not include three each a month apart was insufficient.
Uncertain	Insufficient blood tests to confirm a definite diagnosis of AKI or CKD. This included patients who only had one or two elevated serum creatinine values, a cluster of abnormal tests without evidence of chronicity, and patients with abnormal tests who died before a subsequent fall in creatinine could be determined.
Mild AKI	Patients with a creatinine below the “threshold value” in the previous six months, that subsequently rose above the threshold, but with insufficient change to diagnose AKI (<1.5-fold rise in creatinine or 25% fall in eGFR).

*Note that patients previously categorised as “acute on chronic renal failure” in the original study are also included in this group.

All patients in the cohort had their case notes reviewed by a nephrologist to confirm the classification, ensure that rapid changes in creatinine were not due to RRT, exclude individual results that were implausible, record the cause of AKI and record the clinical location of management. Data were recorded onto a research database by a nephrologist or a research coordinator and every 10^th^ record was cross-checked to confirm accuracy.

### NHS England AKI Algorithm

The first episode of biochemical AKI for each patient, 1^st^ January-30^th^ June 2003, was identified using the NHS England AKI algorithm criteria (at least one of criterion 1–3 in Table 1). For instance, criterion 1 requires an index creatinine to be ≥1.5-fold higher than the median of all creatinine values in the previous 8–365 days. A simplified approach of detecting AKI using the lowest creatinine in 8–365 days was also tested. As sensitivity analyses, to assess if missing baseline data were limiting the performance of the AKI detection algorithm, we constructed a modified (retrospective) algorithm allowing a fourth criterion for AKI—if creatinine fell by a ratio of ≥1.5 over 30 days.

For patients meeting the NHS England definition of AKI, AKI stage was categorised into 3 severity groups based on the magnitude of rise from reference to the highest creatinine within 30 days of first diagnosis (Table 1).

We deviated from the algorithm in two respects. As sample time data were incomplete, we calculated creatinine changes using days rather than hours. Where blood tests were repeated on the same day, we included the highest creatinine. In sensitivity analysis repeat tests on the same day were included but this did not affect our results.

### Patient Characteristics

We recorded the location where AKI was treated (medical ward, surgical ward, intensive care, renal unit, outlying hospital—if not in Aberdeen Royal Infirmary); cause of AKI (sepsis, dehydration, hypotension, hypovolemia, myocardial infarction, nephrotoxins, post-operative, urinary tract obstruction, gastrointestinal haemorrhage, hepatorenal syndrome, myeloma, rhabdomyolysis, pancreatitis, glomerulonephritis, burns) and comorbidities (hypertension, cardiac failure, ischaemic heart disease, diabetes mellitus, peripheral vascular disease, stroke, malignancy, chronic obstructive pulmonary disease, intrinsic renal disease, chronic liver disease). Causes and comorbidities were not mutually exclusive and patients could have more than one recorded.

### Analysis

We summarised the number of patients in each cohort group (RIFLE AKI, mild AKI, uncertain chronicity, CKD) and compared the characteristics of those with RIFLE AKI and CKD.

We compared the number and proportion of patients with AKI alerts generated by the NHS England algorithm in each cohort group. In sensitivity analysis we also reported the number, proportion and timing of patients alerted in each cohort group when the NHS England algorithm was restricted to AKI stages 2 and 3; using a simplified definition for reference creatinine of the lowest creatinine in the previous 8–365 days; and using an extended definition with a retrospective criterion if the creatinine fell over the subsequent 30 days.

Focusing on those with RIFLE AKI, we described how well they were detected by NHS England AKI criteria in subgroups by cause and clinical location. In each subgroup we reported “diagnostic sensitivity” of the NHS England criteria for RIFLE AKI. We have defined diagnostic sensitivity as the number of patients with RIFLE AKI who also met NHS England AKI criteria divided by the total number with RIFLE AKI, expressed as a percentage. We did not evaluate “specificity” as patients with mild AKI or uncertain chronicity may still be true AKI patients.

## Results

### Patient Characteristics

Of 4464 patients, 525 had RIFLE AKI, 449 had mild AKI, 1305 were of uncertain chronicity and 2185 had CKD (Fig 1) as originally diagnosed by a nephrologist. Patients with AKI and CKD were similar in age and sex, but CKD patients had more comorbidities (Table 3).

**Table 3:** Characteristics of the RIFLE AKI and CKD clinical diagnosis groups.

	RIFLE AKI	CKD
Total	525	2185
Male Sex (%)	282 (53.7)	1078 (49.3)
Age ≥70 years (%)	385 (73.3)	1676 (76.7)
Hypertension (%)	146 (27.8)	1184 (54.2)
Congestive cardiac failure (%)	68 (13.0)	321 (14.7)
Ischaemic heart disease (%)	164 (31.2)	818 (37.4)
Diabetes (%)	83 (15.8)	572 (26.2)
Peripheral vascular disease (%)	41 (7.8)	224 (10.3)
Stroke (%)	63 (12.0)	259 (11.9)
Cancer (%)	119 (22.7)	272 (12.4)
COPD (%)	39 (7.4)	128 (5.9)
Dementia (%)	36 (6.9)	76 (3.5)
Intrinsic renal disease (%)	13 (2.5)	71 (3.2)
Liver Disease (%)	15 (2.9)	16 (0.7)
Number of comorbidities (Median, IQR)	2 (1–3)	3 (2–4)

COPD chronic obstructive pulmonary disease, IQR Inter-quartile range

### NHS England Algorithm vs Nephrologist Diagnosis

**Fig 2:**
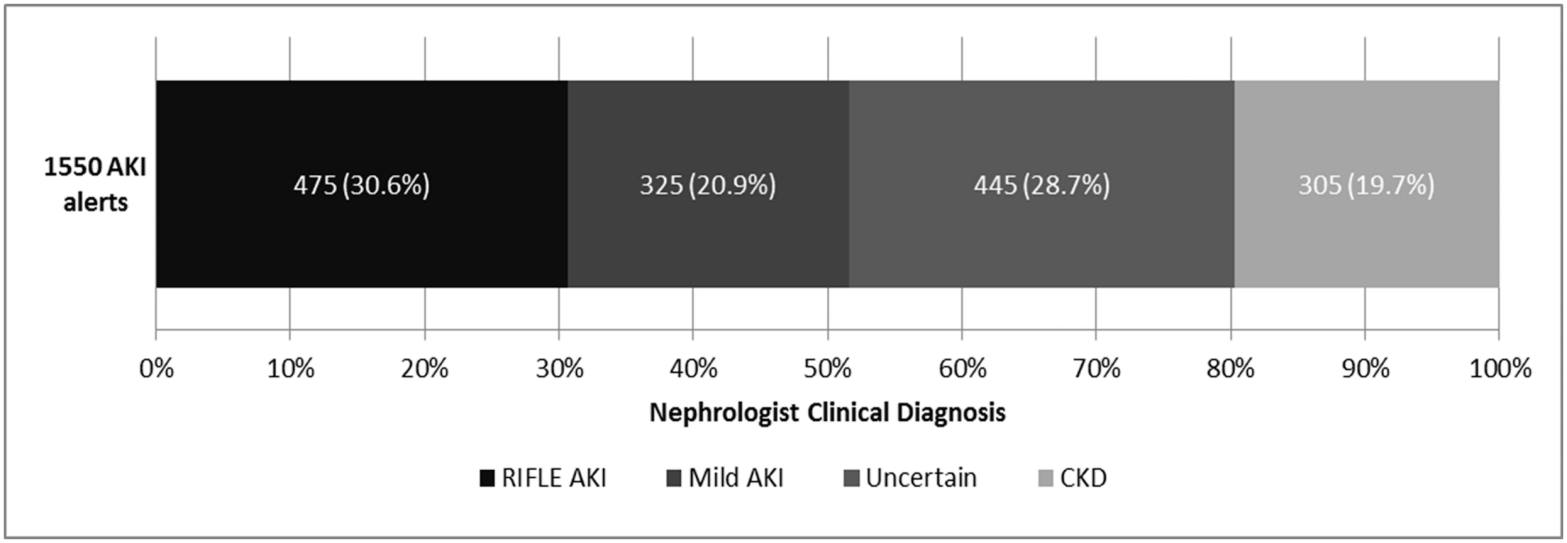
Nephrologist clinical diagnosis of patients who received an AKI alert.

NHS England AKI algorithm criteria alerted 1550 unique patients of which 895 were classified as stage 1, 384 stage 2 and 271 stage 3. Alerts occurred in 475 of 525 (90.5%) patients with nephrologist diagnosis of RIFLE AKI and 325 of 449 (72.4%) patients diagnosed as mild AKI. Alerts also occurred in 305 of 2185 (14%) patients diagnosed by nephrologist as CKD and not AKI. The same 305 CKD patients amounted to 19.7% (305/1550) of all patients alerted (Fig 2).

### NHS England Algorithm vs Nephrologist Diagnosis

**Fig 3:**
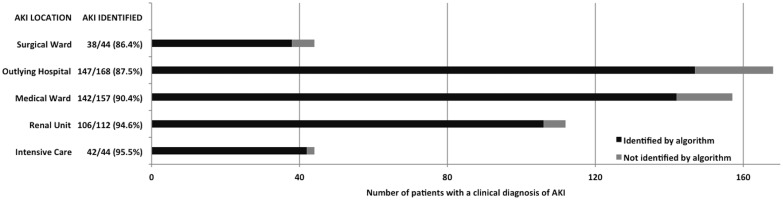
RIFLE AKI patients detected by the NHS England AKI algorithm by clinical location. The greatest proportion of RIFLE AKI patients identified by the NHS England algorithm was in intensive care (95.5%) and the renal unit (94.6%).

**Fig 4:**
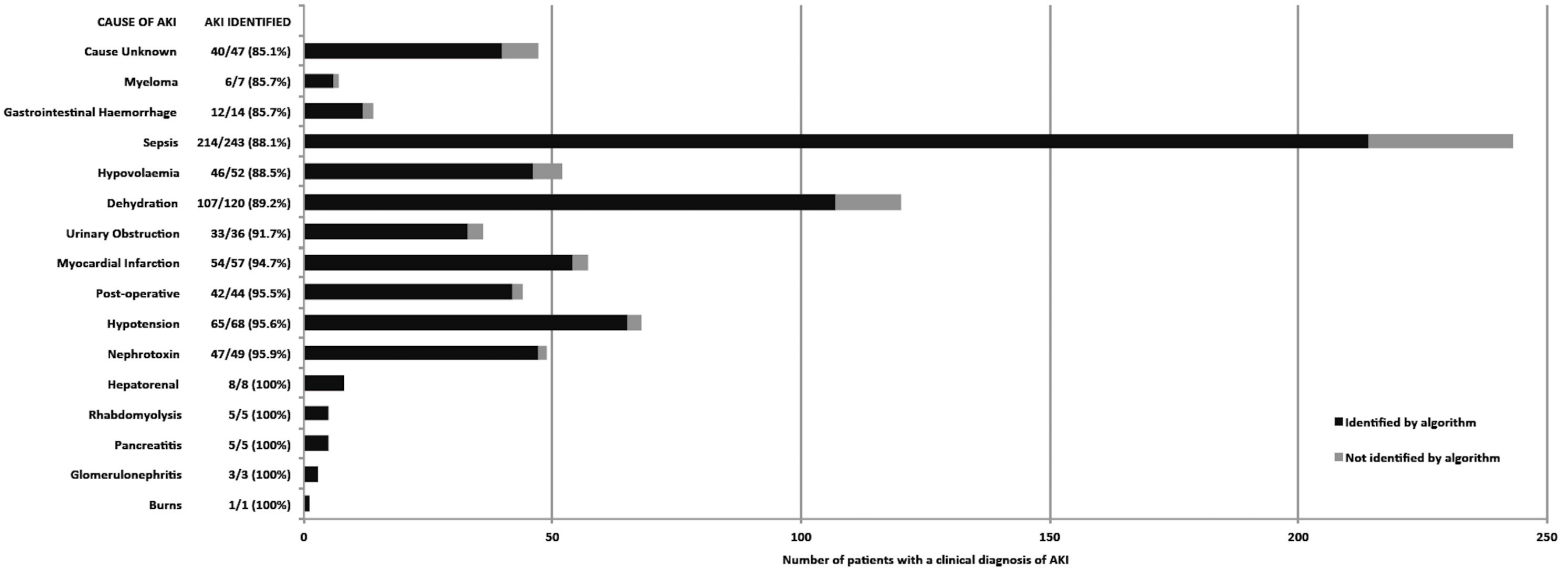
RIFLE AKI patients detected by the NHS England AKI algorithm by AKI cause. Patients could have more than one cause of AKI recorded. The algorithm identified >85% of RIFLE AKI patients across all aetiologies, although it performed least well when the cause of AKI was unknown (85.1%).

Of the 525 patients with RIFLE AKI, a large proportion had sepsis (243, 46.3%) and were on medical or renal wards (325, 61.9%) (Fig 3 and Fig 4). Across locations and AKI aetiologies more that 85% of RIFLE AKI confirmed by a nephrologist was detected, but with some variation.

The algorithm detected almost all RIFLE AKI in intensive care (95.5%) and the renal unit (94.6%), but less AKI in outlying areas (87.5%) and in surgical wards (86.4%). The algorithm also detected RIFLE AKI well across different aetiologies, although least well in patients where the cause of AKI was unknown (85.1%).

### Sensitivity Analyses

**Fig 5:**
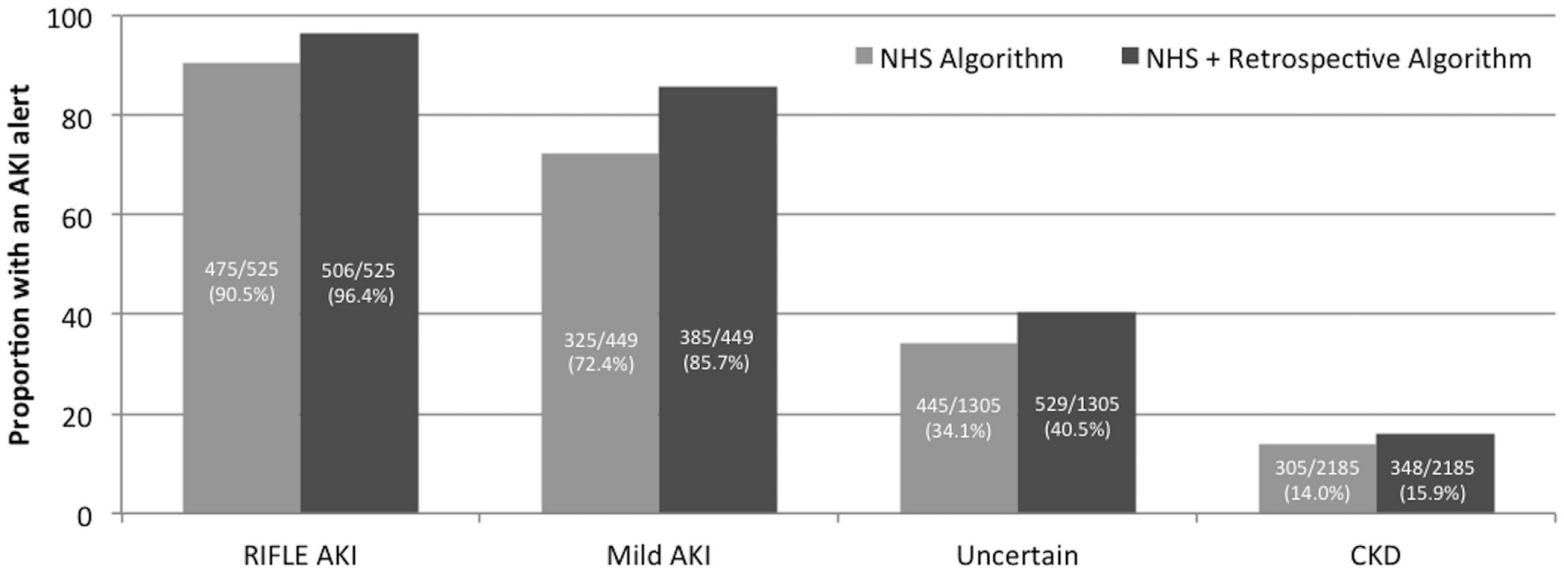
Proportion of patients meeting NHS England and retrospective algorithm criteria. The retrospective AKI algorithm detected additional RIFLE AKI patients to the NHS England algorithm without more alerts in CKD patients without AKI.

To assess if missing baseline data affected AKI detection, a retrospective algorithm was also assessed. This identified additional patients with RIFLE AKI (96.4% vs 90.5%) and mild AKI (85.7% vs 72.4%), with a small increase in the proportion of CKD patients detected as AKI (15.9% vs 14.0%) (Fig 5).

We assessed the consequences of restricting the NHS England algorithm to only stages 2 and 3 AKI alerts. These restricted criteria reduced alerting of CKD patients without AKI to 45 of 2185 CKD patients (2.1%). The restricted criteria, however, also reduced the alerting of RIFLE AKI to 305 of 525 patients (58.1%). In addition, only 173 of 525 (33.0%) RIFLE AKI patients had a stage 2 or 3 alert on their first available alert whereas the other 132 alerted patients had an earlier stage 1 alert. Thus, restriction to stage 2 and 3 AKI alerts would have led to missed, or delayed alerting in 67% of RIFLE AKI patients (Table 4).

**Table 4:** Patients with AKI alerts in each clinical diagnosis group.

	RIFLE AKI	Mild AKI	Uncertain Chronicity	CKD
N	525	449	1305	2185
Alerted by NHS AKI algorithm (%)	475 (90.5%)	325 (72.4%)	445 (34.1%)	305 (14.0%)
Any alert by NHS AKI algorithm of stage 2 or 3 (%)	305 (58.1%)	154 (34.3%)	147 (11.3%)	45 (2.1%)
First alert by NHS AKI algorithm of stage 2 or 3 (%)	173 (33.0%)	111 (24.7%)	106 (8.1%)	28 (1.3%)
Alerted by retrospective algorithm (%)	506 (96.4%)	385 (85.7%)	529 (40.5%)	348 (15.9%)
Alerted by lowest creatinine 8–365 days (%)	462 (88.0%)	349 (77.7%)	536 (41.1%)	448 (20.5%)

AKI acute kidney injury, CKD chronic kidney disease

We also assessed if a combination of three criteria (as in the NHS England algorithm) was necessary or if similar results could be achieved using a more simple single definition. We tested this with a baseline definition of the lowest creatinine in the previous 8–365 days, previously assessed elsewhere [10]. This approach identified 462 of 525 (88.0%) AKI patients, but also 448 of 2185 (20.5%) of CKD patients (Table 4).

## Discussion

This is the first study to compare the NHS England national AKI algorithm with large scale case-note review. We compared a clinical diagnosis from an experienced nephrologist with automated AKI detection using the NHS England criteria. 90.5% of patients with a clinically confirmed RIFLE AKI diagnosis were detectable using the AKI algorithm, and an additional 72.4% of those with “mild” AKI (a rise in creatinine, but insufficient for the older RIFLE criteria) could also be detected. However, patients diagnosed clinically as CKD without AKI were also detected by the automated system (14%) and this represents the necessary trade-off for a sensitive screening measure.

Reassuringly, the NHS algorithm performed well across all clinical locations and aetiologies, consistently detecting more than 85% of RIFLE AKI patients. The best performance in intensive care and the renal unit is consistent with a greater frequency of testing in these patients. Under-detection in surgical patients and outlying hospitals is nevertheless important, as these patients will receive less specialist attention. They may also be more likely to gain from initiatives to improve AKI recognition and guide management. The reason for this reduced performance in surgical patients may be due to less previous outpatient monitoring and fewer baseline data available.

In sensitivity analysis we considered whether the algorithm could be improved further:

First, while the algorithm is automated it is complex to communicate as it involves a combination of three criteria. We therefore tested a simplified approach using only the lowest creatinine in 8–365 days, but the combined NHS England criteria identified more RIFLE AKI patients and fewer CKD patients.

Second, to reduce the burden of “alert fatigue” a strategy of prioritising stage 2 and stage 3 AKI alerts might be considered in some hospitals. We found that prioritising only stage 2 and 3 alerts could reduce over-diagnosis to just 2.1% of CKD patients, but this would involve a sacrifice of missed or delayed alerting in 67% of RIFLE AKI patients that may benefit from early recognition.

Third, we found that RIFLE AKI patients not detected by NHS England criteria could be identified using retrospective detection if creatinine later fell and without increasing over-diagnosis of CKD as AKI. This suggests that missing baseline data may prevent the NHS England algorithm identifying some relevant patients, but a retrospective definition would not be practical for use in real-time where alerts first need to be timely for them to provide benefit. A high creatinine in those without previous results still provides a timely flag prompting monitoring on NHS England criteria, but does not distinguish AKI from CKD.

A major strength of this study is that despite its large size, the cohort is population based rather consisting of referred patients, and every patient was well characterised with their diagnosis and clinical setting confirmed using case-notes. This ensured that patients without AKI who had CKD or were receiving chronic dialysis could be recognised. A limitation is that the clinical diagnosis was evaluated by only one well-experienced nephrologist. This is the first study to compare the NHS England algorithm with a clinical diagnosis. Previous studies have described automated AKI alerts but without assessing diagnostic accuracy[11–13].

Our findings are limited by the clinical practice and different AKI criteria used at the time. Our objective was to compare automated detection with clinical diagnosis, but the two approaches were based on slightly different definitions. In this 2003 cohort, patients were diagnosed clinically using the RIFLE criteria, but the current KDIGO AKI criteria also include smaller creatinine rises (>26 μmol/L rise in creatinine within 48 hours)[1]. This is reflected in the detection of NHS England criteria AKI (based on KDIGO criteria) in 72.4% of those previously labelled “mild AKI”, which will have included patients with these small creatinine rises. In addition, a high threshold creatinine (150 μmol/L for men, 130 μmol/L for women) was used to construct this cohort of patients with renal impairment, but it is possible to have AKI with milder levels of renal impairment. Our findings therefore require careful interpretation as it would still be appropriate for an algorithm to detect AKI in a patient with a clinical diagnosis of “mild AKI” rather than “RIFLE AKI”. On the other hand, the RIFLE AKI diagnosis in this context provides a parsimonious clinical diagnosis of AKI. It is therefore reassuring to demonstrate good diagnostic sensitivity of automated detection for RIFLE AKI, and intuitive for there to be fewer AKI alerts in the indeterminate groups with mild AKI or abnormal results of uncertain chronicity.

Notably, neither the NHS algorithm criteria nor our cohort included measures of oliguria, which is an important component of the current KDIGO criteria, particularly in critical care[1]. These findings and the NHS algorithm therefore must be interpreted with the awareness that only creatinine changes are represented.

Recently a randomised controlled trial using automated alerts for AKI has been reported showing no mortality benefit from alerts versus usual care[14]. We note, however, that the trial used only two of the three NHS England criteria, which may have limited the timing of AKI alerting. Both AKI alert and usual care interventions also took place in the same hospital departments which will have led to contamination between the two study arms. More work is needed to identify not only where AKI is under-detected, but also where and how timely recognition of AKI could best be used to enhance overall care.

In this study the ability of the NHS England algorithm to recognise 90.5% of RIFLE AKI patients and distinguish between patients with AKI and CKD provides support of its value as a clinical adjunct. It is important for the clinician to be aware that not all AKI patients will be detected, although the flag of an abnormally high creatinine may help when absent baseline data may be presumed to be normal. In addition, some CKD patients will be misclassified, but this should not be used as a reason to focus only on severe alerts. Focusing on stage 2 and 3 AKI would reduce misdiagnosis, but would also delay or miss intervention in two-thirds of RIFLE AKI patients who might benefit.

We have shown that automated AKI detection can be effective in detecting AKI across clinical settings, but it must be interpreted carefully as part of an overall clinical assessment. Clinicians should be educated on the importance of interpreting alerts in a clinical context, and proactive review is still critical to pre-empt, diagnose and manage AKI early in patients on outlying wards.
